# Adenosine-5'-Triphosphate (ATP) Protects Mice against Bacterial Infection by Activation of the NLRP3 Inflammasome

**DOI:** 10.1371/journal.pone.0063759

**Published:** 2013-05-22

**Authors:** Yang Xiang, Xuan Wang, Chao Yan, Qian Gao, Sheng-An Li, Jie Liu, Kaifeng Zhou, Xiaolong Guo, Wenhui Lee, Yun Zhang

**Affiliations:** 1 Key Laboratory of Animal Models and Human Disease Mechanisms of the Chinese Academy of Sciences and Yunnan Province, Kunming Institute of Zoology, Chinese Academy of Sciences, Kunming, Yunnan, China; 2 Graduate School of the Chinese Academy of Sciences, Beijing, China; University of California Merced, United States of America

## Abstract

It has been established that Adenosine-5'-triphosphate (ATP) can activate the NLRP3 inflammasome. However, the physiological effect of extracellular ATP on NLRP3 inflammasome activation has not yet been investigated. In the present study, we found that ATP was indeed released during bacterial infection. By using a murine peritonitis model, we also found that ATP promotes the fight against bacterial infection in mice. ATP induced the secretion of IL-1β and chemokines by murine bone marrow-derived macrophages *in vitro*. Furthermore, the intraperitoneal injection of ATP elevated the levels of IL-1β and chemokines in the mouse peritoneal lavage. Neutrophils were rapidly recruited to the peritoneum after ATP injection. In addition, the effects on cytokine and chemokine secretion and neutrophil recruitment were markedly attenuated by the pre-administration of the caspase-1 inhibitor Ac-YVAD-cho. Ac-YVAD-cho also significantly attenuated the protective effect of ATP against bacterial infection. In the present study, we demonstrated a protective role for ATP during bacterial infection and this effect was related to NLRP3 inflammasome activation. Together, these results suggest a role for ATP in initiating the immune response in hosts suffering from infections.

## Introduction

The body’s first line of defense against invading infectious bacteria is provided by the innate immune system [1]. The innate immune system relies on the pattern recognition receptors (PRRs), which include the Toll like receptors (TLRs) and NOD like receptors (NLRs) [2]. The NLRP family proteins are important components of inflammasomes. Inflammasomes are molecular platforms that assemble through the hetero-oligomerization of NLRP, ASC and pro-caspase-1. Upon the assembly of this macro-molecular platform, pro-caspase-1 is activated and subsequently processes proinflammatory cytokines such as IL-1β and IL-18 [3]. Recent studies have found that inflammasomes, especially the NLRP3 inflammasome, plays a pivotal role in fighting bacterial infections [4]. The absence of caspase-1 or other inflammasome components leads to increased susceptibility to *S. typhimurium* infection [5]. *in vivo* studies with caspase-1^–/–^ mice have revealed a high susceptibility to *B. pseudomallei* challenge, which can cause melioidosis [6].

Numerous studies have demonstrated that extracellular ATP plays important roles in the immune system. The autocrine production of ATP by neutrophils amplified neutrophil chemotaxis [7]. Extracellular ATP can also induce interleukin 8 secretion by human urinary tract cells in a P2Y receptor activation-dependent manner [8]. Extracellular ATP was able to enhance interleukin-6 transcription [9]. Furthermore, ATP could provide a costimulatory signal to T cells and drive the differentiation of intestinal T helper 17 cells [10]. ATP has also long been considered an inducer of NLRP3 inflammasome [11]. The activation of the NLRP3 inflammasome in response to extracellular ATP is mediated by the purinergic receptor P2X7. Under conditions of high extracellular ATP concentrations, p2x7 is activated, which my in turn induce the formation of large pores by pannexin-1, followed by potassium efflux and NLRP3 inflammasome activation, leading to IL-1β maturation and secretion [12]. ATP is exceptionally abundant in all cell types, but usually does not exist extracellularly under normal circumstances. ATP may be released upon infection or other danger stimulation, and extracellular ATP may then become an alarm signal that can initiate innate immunity, likely by activating the NLRP3 inflammasome. Because of these characteristics, ATP is generally considered an endogenous danger signal [10]. Consistent with this idea, ATP is released by monocytes upon stimulation with pathogen-sensing receptor ligands, and it subsequently induces interleukin 1β and interleukin 18 in an autocrine manner [13]. ATP can also be derived from bacteria [14], raising the possibility that ATP may protect animals against bacterial infection. In fact, previous studies have revealed an important role for ATP and P2X7 receptor in protecting against Chlamydia infection in vaginally infected mice [15], [16]. P2X7 also plays an important role in LPS-induced lung injury *in vivo* [17].

Neutrophils are the most common type of white blood cell, comprising approximately 50 to 70 percent of all white blood cells. They are the first type of immune cells to arrive at the site of infection by chemotaxis [18]. Studies have revealed that the inflammasome-mediated production of IL-1β is required for neutrophil recruitment in response to *Staphylococcus aureus in vivo* [19]. The mouse peritonitis model has long been used to study the effects of antibiotics *in vivo* [20]. In addition, this model is quite useful for studying anti-infection immunity.

Based on the characteristics of ATP, we hypothesized that it would effectively protect mice from bacterial infection, and this effect might be related to NLRP3 inflammasome activation. In the present study, we tested this hypothesis in a mouse peritonitis model. We found that intraperitoneal injection of ATP significantly accelerated bacterial clearance, thus reducing the mortality rate in the mouse peritonitis model. This effect was dependent on NLRP3 inflammasome activation and neutrophil recruitment.

## Materials and Methods

### Ethics Statement

This study was performed in strict accordance with the recommendations of the Guide for the Care and Use of Laboratory Animals of the National Institutes of Health. The protocol was approved by the Committee on the Ethics of Animal Experiments of the Kunming institute of Zoology (Permit Number: 201112008). All efforts were made to minimize suffering. I state that humane endpoints were used in our survival studies. The mice were monitored every 6 hours, and if the animal were not able to roll over from side to chest, they were humanely euthanized by CO_2_ inhalation.

### Mice, Cell Lines and Bacteria Strains

The mouse strain used in the present study was “Kunming mice”. The Kunming mice were purchased from the experimental animal center of Kunming Medical School. Four-week-old male animals with Weights of 18–22 g were used in all experiments. Generally, the experimental groups were composed of at least six mice. Animals from the same experimental group were kept in the same cage under constant temperature (22°C) and humidity with a 12-h light/dark cycle and had access to food and water ad libitum throughout the study. The mice were euthanized by CO_2_ inhalation at the end of the experiments. All procedures, care and handling of animals were approved by the Ethics Committee of the Kunming Institute of Zoology at the Chinese Academy of Sciences.

Primary murine bone marrow-derived macrophages (BMDM) were isolated from the femurs of mice and cultured as described by Riteau, N. et al [21]. Murine neutrophils were isolated as previously described [7], [22]. Briefly, the mice were exsanguinated into PBS containing 10 mM EDTA. The blood cells were obtained by centrifugation. The cells were then resuspended and layered onto a Percoll gradient of 80%, 70% and 50% in PBS. After centrifugation, the cells at the 80% and 70% interface were collected. The red blood cells were eliminated by hypotonic lysis.

The bacterial stains were obtained from the First Affiliated Hospital of Kunming Medical College (China) and belonged to two different species, as follows: (i) *E.coli* ATCC 25922, a clinically isolated *E.coli* strain (producing extended-spectrum beta-lactamase and resistant to I, II, III and IV generation cephalosporins) [23] and (ii) *S. aureus* ATCC 25923, a clinically isolated *S. aureus* strain.

### ATP Assay

BMDMs cultured in 12-well plates were washed three times with PBS and exposed to serum-free medium containing bacteria for the indicated time periods. At the indicated time point, the culture medium was harvested and centrifuged for 10 min at 12000 rpm. The ATP concentration in the supernatant was assayed using a highly sensitive luciferase-based technique (Sigma). The luciferase activity was measured on a luminometer (Tecan) and compared with an ATP standard. For assaying the ATP levels *in vivo*, the concentration in mouse peritoneal lavage fluid was assessed as previously described.

### Western Blotting

BMDMs grown in 12-well plates were treated with ATP for the indicated time periods. The cells were then collected, washed, and immediately lysed on ice in lysis buffer containing 50 mM HEPES (pH 7.4), 5 mM EDTA, 50 mM NaCl, 1% Triton X-100, 50 mM NaF, 5 mg/mL aprotinin, 5 mg/mL leupeptin and 1 mM phenylmethylsulfonyl fluoride. The Proteins (30 µg) were electrophoresed on SDS–polyacrylamide gels and transferred onto PVDF membranes. The membranes were subsequently blocked with 3% BSA and incubated with the appropriate primary and secondary antibodies. The protein bands were visualized with Super Signal chemiluminescence reagents (Pierce, Rockford, IL, USA), as previously described [24].

### *In vitro* Chemotaxis Assays

*In vitro* neutrophil chemotaxis was assessed using Millicell filters with 3-µm pores (Corning) which were pre-incubated with 10% FBS in RPMI 1640, inserted in 24-well plates, and washed twice with serum-free RPMI 1640 before use. 600 µl of different conditioned medium were added to the bottom of the chamber. 5×10^5^ neutrophils in 100 µl were added to the top of the chamber. The plates were incubated at 37°C/5% CO_2_ for 2 hours. The cells in the bottom were counted with a hemacytometer.

### Induction of Peritonitis

Peritonitis was induced as described by Rosemarijn Renckens et al [25]. Briefly, the bacteria was cultured in Luria-Bertani medium at 37°C, harvested at the mid-log phase, and washed twice with sterile saline before injection. The mice were injected intraperitoneally with the appropriate amount of bacteria in a 150 µl sterile saline. Mortality observations were made every 12 hours. To assessing the protective role of ATP, it was injected 1, 4 or 24 hours before bacteria injection.

### Peritoneal Lavage Harvesting

At the time of sacrifice, the mouse abdomen was first sterilized by 75% ethanol. A peritoneal lavage was then performed with 2 ml of sterile PBS using an 18-gauge needle, and the peritoneal lavage fluid was collected in sterile tubes. The collected peritoneal lavage fluid was directly used for bacterial counting. After centrifugation, the supernatant was used to assess the concentration of cytokines or ATP, and the cells were subjected to flow cytometric analysis.

### Peritoneal Bacterial Counting

The peritoneal lavage was harvested and collected in sterile tubes and eight serial 10-fold dilutions were made for each sample of the homogenates. 100 µl of each dilution was plated onto blood agar plates. The plates were incubated at 37°C for 20 hours, and the CFU were counted and corrected for the dilution factor. The threshold value of the method was 100 CFU. Dead animals were assigned the highest CFU count obtained in the experiment.

### Cytokine Determination

Peritoneal interleukin-1β, KC and MIP-2 levels in the mice were determined using commercial ELISA kits (purchased from R&D Systems) according to the manufacturer’s instructions.

### Flow Cytometry Analysis

Peritoneal exudate cells (PEC) were washed with 1640 medium and blocked with 5% normal mouse serum for 10 min at room temperature. After the cells were washed with 1640 medium, FITC-conjugated anti-Gr-1 (Biolegend) and PE-conjugated anti-F4/80 (Biolegend) were added, and the cells were incubated at room temperature for 15 min. The cells were then analyzed on a FACS Calibur flow cytometer (Becton-Dickinson, San Jose, CA), and FlowJo softerware was used to analyze the data.

### Statistical Analysis

Survival data were analyzed using the Kaplan–Meier method, and survival curves were compared using the log-rank test in univariate analysis. All the other data were presented as the means±SEMs. Two-sample comparisons were performed using Student’s t-tests.

## Results

### Bacteria-infected Macrophages Release ATP *in vitro* and Bacteria Stimulate ATP Release from Peritoneal Cells *in vivo*

**Figure 1:**
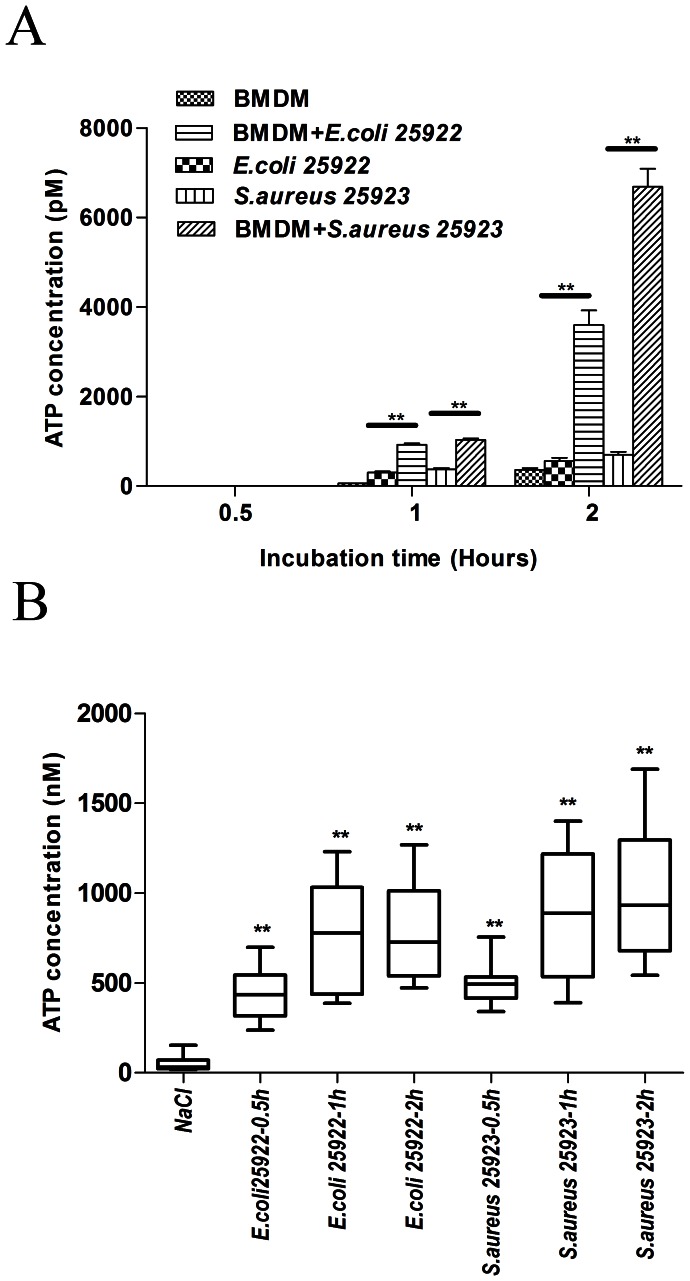
ATP was released after infection both *in vitro* and *in vivo*. (**A**) *In vitro* analysis of ATP released from BMDMs and bacteria. The ATP levels in the culture medium from wells containing only cells, only bacteria (3×10^8^ CFU/ml) or both were assessed after 0.5, 1 and 2 hour of incubation. The data are presented as the means±SEMs (n = 5). (**B**) *In vivo* analysis of ATP release from murine peritoneal cells. Mice received intraperitoneal injections of E.coli 25922 (3×10^8^ CFU per mouse) or S. aureus 25923 (3×10^8^ CFU per mouse). The mice were sacrificed 0.5, 1 or 2 hours after injection. The peritoneal lavage fluid was harvested by injecting 2 ml of PBS into the peritoneum. The ATP concentration in the fluid was then assayed. As a negative control, 150 µl of 0.9% NaCl was intraperitoneally injected, and the peritoneal lavage fluid was harvested 2 hours after NaCl injection. The data are presented as the means±SEMs (n = 10). Values that are significantly different different are indicated by asterisks as follows: **, P<0.01.

A number of studies have reported that bacteria-infected cells were able to release ATP [8]. In addition, ATP was also released during bacterial growth. In the present study, the ATP levels in the culture medium from uninfected cells, bacteria or infected cells were assessed using a bioluminescent assay. After a 0.5-hour incubation, ATP was barely detectable in all three types of culture media. However, after 1 hour or 2 hours of incubation, the ATP concentration in the culture medium from the bacteria-infected cells was markedly increased compared with that of the uninfected cells and bacteria (**Figure 1A**). Although the uninfected cells alone and the bacteria also released ATP, the quantity of ATP released by the bacteria-infected cells was much larger (more than 5-fold). These data suggested that ATP was released during infection *in vitro*.

*In vivo* analysis revealed that bacteria infection promoted ATP release by the cells at the site of infection (**Figure 1B**). The ATP concentration in the peritoneal lavage fluid was significantly elevated 0.5, 1 and 2 hours after either *E.coli* or *S.aureus* infection. These results suggested that ATP may play a physiological and pathological role during bacterial infection.

### ATP Induces Secretion of Interleukin 1β, KC and MIP-2 from Bone Marrow Derived Macrophages (BMDMs) *in vitro* in a Caspase-1 Activation-dependent Manner

**Figure 2:**
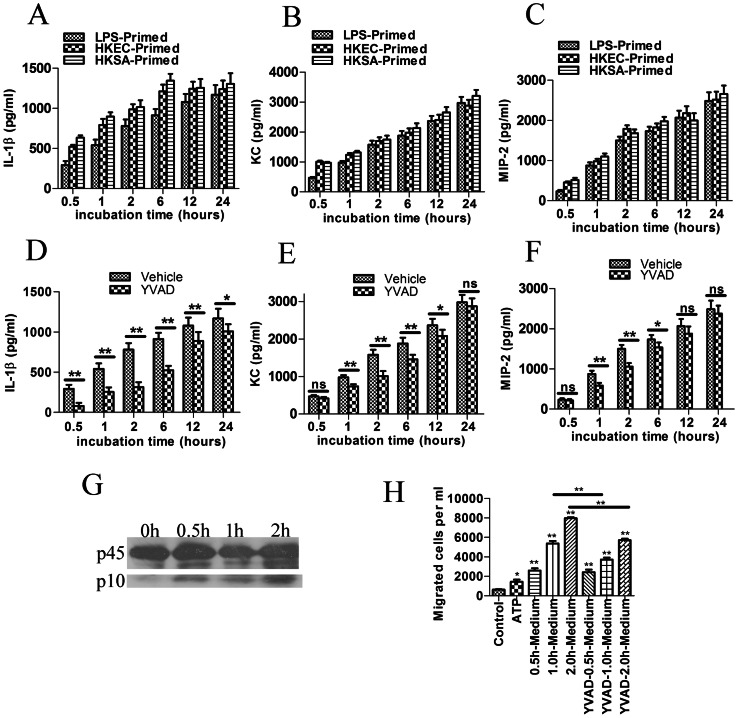
ATP stimulats cytokine and chemokine secretion and inflammasome activation directly and indirectly induces ***in vitro*** **neutrophil chemotaxis.** (**A, B and C**) BMDMs were primed with LPS (500 ng/ml), heat killed *E. coli* 25922 (HKEC) (3×10^8^ CFU/ml) or heat killed *S. aureus* 25923 (HKSA) (3×10^8^ CFU/ml) and the medium was replaced with fresh serum-free medium and incubated with ATP (2 mM) for 0.5, 1, 2, 6, 12 and 24 hours. The levels of IL-1β, KC and MIP-2 were then assessed by ELISA. (**D, E and F**) To test the role of caspase-1 in the cytokine secretion, the cells were pre-incubated with the Caspase-1 inhibitor Ac-YVAD-cho (50 µM) for 30 min after LPS-priming, then incubated with ATP. The data are presented as the means±SEMs (n = 5). Values that are significantly different are indicated by asterisks; *(P<0.05), **(P<0.01). Values that showed no significant differences are marked “ns”. (**G**) BMDMs incubated with ATP (2 mM) for the indicated times were lysed and immunoblotted with Caspase-1 antibody (Santa Cruz). The appearance of the p10 subunit represented the activation of caspase-1 and the inflammasome. The presented graph is representative of at least three independent experiments. (**H**) ATP-stimulated culture medium was harvested, and the ability to induce neutrophil chemotaxis was assessed using 3-µm-pore transwells. The data are presented as the means±SEMs (n = 5). The number of migrated cells was compared with the control (containing fresh medium in the lower chamber), and the values that are significantly different are indicated by asterisks as follows: *(P<0.05), **(P<0.01).

To assess the influence of ATP on cytokine and chemokine secretion, cells were primed with LPS (500 ng/ml), heat killed *E.coli* 25922 (3×10^8^ CFU/ml) or heat killed *S. aureus* 25923 (3×10^8^ CFU/ml), and the medium was replaced with fresh serum-free medium. The cells were then incubated with ATP (2 mM) for 0.5, 1, 2, 6, 12 and 24 hours. After priming, the ATP treatment strongly induced Interleukin 1β, KC and MIP-2 secretion (**Figure 2A–C**). Cells that were preincubated with the caspase-1 inhibitor Ac-YVAD-cho (50 µM) secreted markedly less Interleukin 1β, KC and MIP-2 (Figure 2D–F). The Interleukin 1β level was reduced more than 50% after 0.5, 1, 2 and 6 hours of incubation. However, when the incubation period was prolonged to 12 or 24 hours, the inhibitory effect of Ac-YVAD-cho was much weaker. Caspase-1 inhibition did not influence the KC and MIP-2 levels after 0.5 hours of incubation with ATP. However, caspase-1 inhibition attenuated KC and MIP-2 secretion after incubation with ATP for 1, 2, and 6 hours. Ac-YVAD-cho did not inhibit secretion after 12 or 24 hours of incubation for KC and 24 hours for MIP-2. These data indicated that ATP induced interleukin 1β, KC and MIP-2 secretion dependent, at least partially on caspase-1 activation during the early phase. The cells were also lysed for western blotting to detect the p10 subunit of caspase-1, thus assessing the level of caspase-1 activation. Caspase-1 was activated after 0.5 to 2 hours of incubation with ATP, and the activation level became stronger with prolonged incubation (**Figure 2G**).

### ATP-conditioned BMDM Culture Medium Promoted Neutrophil Chemotaxis *in vitro*

Because ATP induced chemokine (KC and MIP-2) secretion by BMDMs, we inferred that ATP-conditioned BMDM culture medium might promote neutrophil chemotaxis. This idea was tested using a transwell model. The results demonstrated that although ATP alone could slightly enhance neutrophil migration (approximately twice the control), ATP-conditioned medium enhanced neutrophil chemotaxis to a much greater extent (eight fold compared with the control). Ac-YVAD-cho pretreated ATP conditioned medium showed attenuated the ability to induce neutrophil chemotaxis (**Figure 2E**).

### ATP Protects Mice Against Bacterial Infection *in vivo*

**Figure 3:**
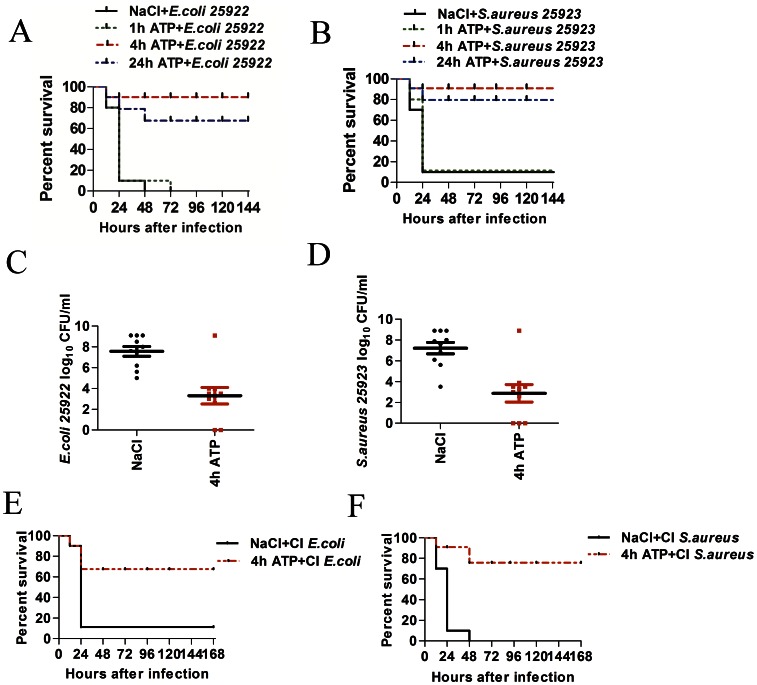
ATP protected mice from bacterial infection. (**A**) The pre-injection of ATP (50 mg/kg) 1, 4, or 24 hours before bacterial challenge *(E. coli*, ATCC25922, 2×10^8^/mice) manifested different potentials to protect mice against bacterial infection (n = 10, P<0.05 by log-rank test). (**B**) The pre-injection of ATP (50 mg/kg) 4 hours before bacterial challenge (*S. aureus*, ATCC25923, 5×10^8^/mouse) manifested a different ability to protect mice from bacterial infection (n = 10, P<0.05 by log-rank test). (**C**) Bacterial counts in the mouse peritoneum 12 hours after the bacterial (E. coli, ATCC25922, 2×10^8^/mice) challenge (n = 10, P<0.05 by t test). Dead animals were assigned the highest CFU count obtained in the experiment. ATP pre-administration (4 hours before bacterial challenge) markedly reduced the bacteria counts in the mouse peritoneum. (**D**) Bacterial counts in the mouse peritoneum 12 hours after the bacterial (*S. aureus*, ATCC25923, 5×10^8^/mouse) challenge (n = 10, P<0.05 by t test). Dead animals were assigned the highest CFU count obtained in the experiment. ATP pre-administration (4 hours before bacterial challenge) markedly reduced the bacterial counts in the mouse peritoneum. (E) Pre-injection of ATP (50 mg/kg) 4 hours before the bacteria challenge (E. coli, clinically isolated, 2×10^8^/mouse) reduced the mortality rate from 90% to 40% (n = 10, P<0.05 by log-rank test). (F) Pre-injection of ATP (50 mg/kg) 4 hours before the bacteria challenge (*S. aureus*, clinically isolated, 5×10^8^/mice) reduced the mortality rate from 100% to 20% (n = 10, P<0.05 by log-rank test).

As a generally accepted danger signal, ATP can activate the NLRP3 inflammasome and thus enhance the production of pro-inflammatory cytokines, such as interleukin 1βand chemokines, such as KC and MIP-2. Based on its characteristics and the above *in vitro* results, we hypothesized that ATP could initiate innate immunity and thereby protect mice from bacterial infection *in vivo*. To assess the protective effect of ATP against bacterial infection, mice were injected intraperitoneally with 0.1 mL ATP (1 mg per mouse) in saline 1, 4 or 24 hours prior to a challenge with various types of bacteria. The results showed that the administration of ATP 4 hours or 24 hours prior to the challenge indeed significantly increased the survival rate of the infected mice regardless of the type of bacteria (**Figure 3**). The mice infected by either gram-positive (*S. aureus*) or gram-negative (*E. coli*) bacteria could be efficiently protected by pre-administration of ATP (**Figure 3**). Infection caused by clinically isolated *E.coli* (resistant to I, II, III and IV generation cephalosporins) (Figure 3E) and oxacillin resistant S. aureus (Figure 3F) could also be efficiently prevented (ATP pre-administration 4 hours before challenge). The bacterial counts in the peritoneum of mice that received the ATP treatment (4 hours before challenge) were greatly reduced compared with the control group (**Figure 3C** **and** **Figure 3D**). Together, these results demonstrated that ATP was indeed able to protect mice from bacterial infection.

### ATP Induces the Secretion of IL1β, KC and MIP-2 and Neutrophils Recruitment *in vivo*

**Figure 4:**
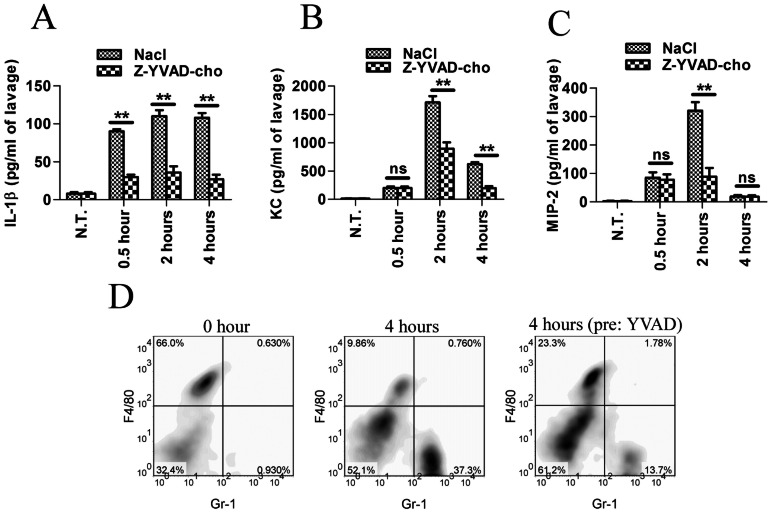
The ATP-induced cytokine and chemokine secretion and neutrophil recruitment *in vivo* are partially dependent on caspase-1 and inflammasome activation. (**A, B and C**) ATP (50 mg/kg) injection promoted the secretion of IL-1β, KC and MIP-2. Ac-YVAD-cho (5 mg/kg) attenuated the secretion of IL-1β, KC and MIP-2 induced by ATP. The data are presented as the means±SEMs (n = 6). The values that are significantly different are indicated by asterisks; **(P<0.01). The values with no significant differences were marked as “ns”. (**D**) Peritoneal exudate cells were stained and analyzed. Gr-1^low^F4/80^high^ cells represent peritoneal monocytes and Gr-1^high^ F4/80^low^ cells represent recruited neutrophils. The data are representative of three independent experiments.

ATP possesses protective effect against bacterial infection in the mouse model, but it cannot directly kill bacteria. Therefore, the only way for it to assert this role is to modify the host immune response. ATP is generally accepted as the inducer of the NLRP3 inflammasome. Moreover, the above *in vitro* experiments demonstrated that ATP can enhance cytokine and chemokine secretion and neutrophil chemotaxis. Thus, in the present study, after ATP stimulation, the peritoneal lavage was harvested, and the cytokines were quantified using specific ELISA assays. The results indicated that the peritoneal IL-1βlevels were markedly elevated even only 30 minutes after ATP injection. The IL-1βlevels were nearly stable (approximately 120pg/ml) between 2 hours and 6 hours after ATP administration (Figure 4A). We also determined the KC and MIP-2 levels in the mouse peritoneum after ATP stimulation. These results demonstrated that both the KC and MIP-2 concentrations in the peritoneal lavage were strongly elevated after ATP stimulation (Figure 4B and Figure 4C). The two chemokines were slightly increased 30 minutes (203 pg/ml for KC, 85 pg/ml for MIP-2) after ATP stimulation, and reached the highest levels 2 hours post-administration (1713 pg/ml for KC, 320 pg/ml for MIP-2) before decreasing. Six hours after ATP injection, the KC and MIP-2 concentrations decreased to 621 pg/ml and 19 pg/ml, respectively. The pre-injection of the caspase-1 inhibitor strongly reduced the level of IL-1βat 0.5, 2 and 4 hours after ATP injection. However, the influence of the Caspase-1 inhibitor on KC and MIP-2 secretion was dependent on the amount of time after ATP injection. The presence of the capsase-1 inhibitor did not influence KC and MIP-2 after short-term ATP stimulation (0.5 hour). However, the presence of the caspase-1 inhibitor strongly reduced the levels of these cytokine and chemokines 2 hours after ATP injection. The caspase-1 inhibitor reduced the KC levels but not MIP-2 levels 4 hours after ATP injection.

KC and MIP-2 are traditional neutrophil chemotactants. Neutrophils are very important in the initial phase of infection [26]. Because ATP strongly induced KC and MIP-2 after ATP stimulation, we predicted that neutrophils might flux into the peritoneum of mice that were injected with ATP. This prediction was confirmed by double staining for F4/80 and Gr-1. Before ATP injection, less than 1 percent Gr-1 positive neutrophils were detected (Figure.4D left). However, the neutrophils rapidly increased 4 hours after intraperitoneal administration of ATP, when the percentage of neutrophils in the mouse peritoneum was 37.3% (Figure 4D Middle). These data suggested that ATP might strongly induce the recruitment of neutrophils into the inflamed area of the body. The pre-injection of the caspase-1 inhibitor attenuated the influx of neutrophils by reducing the percentage of neutrophils from 37.3% to 13.7%, which may suggest that influx of neutrophils is dependent on caspase-1 and inflammasome activation.

### The Protecting Effect is Partially Dependent of NLRP3 Inflammasome Activation

**Figure 5:**
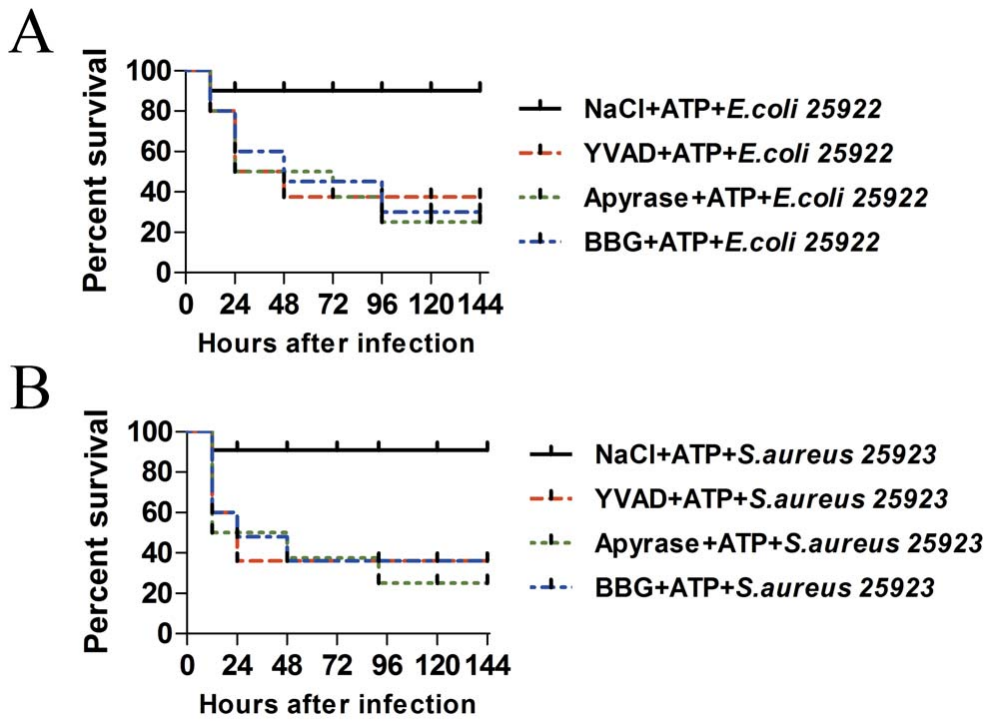
ATP degradation, P2X7 inhibition and Caspase-1 and inflammasome inhibition attenuated the protective effect of ATP against bacterial infection. (**A**) YVAD (5 mg/kg), Apyrase (50 U/kg) or BBG (60 mg/kg) pre-injection prior to ATP reduced the survival rate of the mice infected with *E. coli* 25922 (n = 10, P<0.05 by log-rank test). (**B**) YVAD (5 mg/kg), Apyrase (50 U/kg) or BBG (60 mg/kg) pre-injection before ATP reduced the survival rate of mice infected with *S. aureu*s 25923 (n = 10, P<0.05 by log-rank test).

To confirm that the protective effect was exerted by ATP but not its degraded products ADP or adenosine, apyrase (50 U/kg) was intraperitoneally injected 1hour before ATP administration. The results demonstrated that although apyrase was not able to completely abolish the protective effect of ATP, it indeed strongly attenuated the effect (Figure 5). We also tested whether P2X7 receptor participated in the protective role. The P2X7 receptor antagonist Brilliant Blue G (BBG, 60 mg/kg) was also intraperitoneally injected 1 hour before ATP administration. Consistent with our inference, BBG attenuated the protective role of ATP against infection. We next investigated whether the protective effect of ATP against bacterial infection was dependent on the activation of NLRP3 inflammasome. To this end, the caspase-1 inhibitor ac-YVAD-cho (5 mg/kg) or vehicle were intraperitoneally injected 1 hour before the administration of ATP [27]. The results showed that YVAD markedly attenuated the increased level of IL1β, KC and MIP-2 induced by ATP (Figure 4A, Figure 4B and Figure 4C). YVAD also strongly reduced the ATP-induced neutrophil recruitment (Figure 4D). Based on these results, we can reasonably infer that the inhibition of inflammasome activation by the caspase-1 inhibitor may weaken the protective effect of ATP against bacterial infection. Thus, we tested whether the protective effect of ATP would be changed by administering YVAD before ATP injection. Consistent with our prediction, YVAD administration before ATP significantly reduced the survival of mice compared with those mice that only received ATP injection, and the detailed results are were shown in **Figure 5**. These results together indicated that the protection against bacterial infection is dependent on NLRP3 inflammasome activation by ATP and the subsequent activation of caspase-1.

## Discussion

As a newly identified pattern recognition receptor (PRR) protein complex, inflammasomes have been considered to be excellent sensors in the response to a broad spectrum of PAMPs and DAMPs [28]. Inflammasomes play important roles in the host defense against microbial infection [29]. Among them, the NLRP3 inflammasome has been the most extensively researched in recent years. The NLRP3 inflammasome can be activated by a wide range of pathogen-derived stimuli, such as pore-forming toxins [30]. Interestingly, some antibiotics such as neomycin, polymyxin B, Gramicidin and tyrothricin are also able to activate the NLRP3 inflammasome [31]. Antifungal drugs, such as amphotericin B, nystatin, and natamycin, are also able to activate the NLRP3 inflammasome [32]. These findings suggest that some antibiotics and antifungal drugs not only kill the invading microorganisms but also modulate innate immunity through NLRP3 inflammasome activation. ATP is a typical inducer of the NLRP3 inflammasome. Based on the above characteristics of ATP, it is naturally inferred that extracellular ATP may protect mice from bacterial infection *in vivo*. Moreover, ATP induced inflammasome activation may have physiological significance because ATP can be derived from both the infected host cells and the pathogens when the host is suffering infection. Based on our knowledge of inflammasome and ATP, we hypothesized that ATP pre-administration might enhance the host immune response, thus increasing the host survival rate. The present study is just based on this hypothesis.

We first analyzed the presence of ATP during infection both *in vitro* and *in vivo*. Our *in vitro* results are consistent with studies from another group [8]. In addition, the results of the *in vivo* experiment are promising, because intraperitoneal bacterial infections induced ATP release. The results remind us that ATP has physiological and pathological roles during bacterial infection.

**Figure 6:**
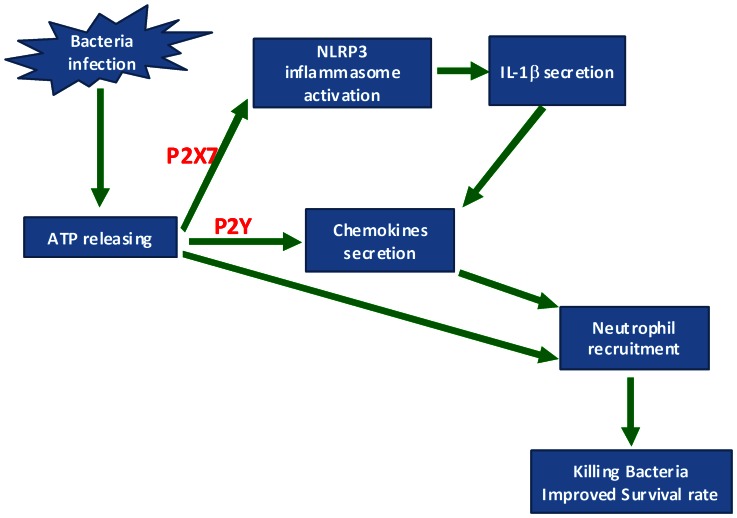
A model for the role of ATP in facilitating the host defense against bacterial infection. During bacterial infection, ATP was released by both the infected cells and the bacteria. The released ATP is able to facilitate neutrophil recruitment to the site of infection by three potential mechanisms: (1) directly enhancing neutrophil chemotaxis; (2) inducing chemokine secretion from resident macrophages through the activation of P2Y receptors; (3) inducing NLRP3 inflammasome activation and Interleukin-1β secretion through the P2X7 receptor, while Interleukin-1β further enhances chemokine secretion. The early recruitment of neutrophils facilitated the clearance of bacteria and the survival rate in mice.

The *in vitro* results indicated that ATP finally induced neutrophil chemotaxis. The neutrophil chemotaxis was a combined effect induced directly by ATP and indirectly by chemokines such as KC and MIP-2. KC and MIP-2 were also induced both directly by ATP and indirectly by IL-1β. Because a caspase-1 inhibitor markedly inhibited the secretion of not only IL-1β but also KC and MIP-2, we can infer that secondary stimulation by IL-1βstrongly contributed to KC and MIP-2 secretion. The present study suggests that the chemokine secretion and neutrophil chemotaxis induced by ATP *in vitro* is a complicated effect (Figure 6).

Based on the characteristics and the *in vitro* studies of ATP, we tested the initial hypothesis that ATP may protect the host against bacterial infection by using a mouse acute peritonitis model. In mice, death due to peritonitis was induced by the intraperitoneal injection of bacteria. Our results clearly showed that the injection of ATP 4 hours before infection indeed reduced the mortality in mice, which was consistent with the prediction. For the first time, this study directly demonstrated that the elevation of extracellular ATP could benefit the host defense against bacterial infection *in vivo*.

To reveal the possible mechanism by which ATP protects mice against bacterial infections, the IL-1β, KC and MIP-2 levels were assessed in mouse peritoneal lavage fluids. IL-1β production in the mouse peritoneum was an inevitable result of NLRP3 inflammasome activation by extracellular ATP. A previous study revealed that IL-1β was able to induce the secretion of C-X-C chemokines, such as MIP-2 and KC [33], and subsequent neutrophil influx. Thus, we assessed the KC and MIP-2 levels in the mouse peritoneum. Consistent with the *in vitro* results, these two chemokines, which are the major neutrophil chemoattractants [34], were markedly elevated. Consequently, neutrophils were recruited to the mouse peritoneum. It is generally accepted that neutrophils are important for the host to fight infection [26], [35]. The recruitment of neutrophils into the peritoneum appeared to be beneficial to facilitate the clearance of invaded bacteria. The rapid recruitment of neutrophils was not mainly due to a direct effect of ATP. Another report has mentioned that ATP was a monocyte chemoattractant, with only a moderate influence on neutrophils [36]. The results from the *in vivo* cytokine and chemokines assessments were consistent with the results from the *in vitro* research.

Previously, Tschopp stated that the physiological significance of extracellular ATP-mediated NLRP3 inflammasome activation is controversial [2]. The doubts are based on the following two reasons: (1) high concentrations (2–5 mM) are needed for the *in vitro* activation of the NLRP3 inflammasome [11], and (2) rapid hydrolysis by ectonucleotidases occurs *in vivo* [37]. However, the concentration of ATP determined by researchers is the average concentration in the medium (*in vitro*) and body fluid (*in vivo*). For a short time (several minutes) after ATP secretion, its concentration around the cell may be very high. The secreted ATP may act on the cell in an autocrine manner to activate the NLRP3 inflammasome. It is logical that NLRP3 activation requires a high concentration of ATP, thus restricting the ATP-induced pro-inflammatory effect to limited region in the body. In this study, we injected ATP intraperitoneally before a bacterial challenge. This raises the problem that the protective effect may be exerted by the degradation products, such as adenosine. In fact, adenosine did protect against sepsis-induced mortality by coupling its receptor A2B [38], [39]. Thus, apyrase, which can degrade ATP, and Brilliant Blue G, which can antagonize the P2X7 receptor, were injected before ATP injection, and both apyrase and BBG attenuated the protective effect of ATP. These results indicate that ATP and its receptor P2X7 play a central role in protecting mice from bacterial infection. In addition, a pharmacological inhibitor, AC-YVAD-cho, which selectively inhibits the activation of Caspase-1, was used to reveal the role of NLRP3 inflammasome activation in protecting mice from bacterial infection. AC-YVAD-cho administration indeed markedly attenuated the protective effect of ATP. Thus, we demonstrated that the protective effect of ATP against bacterial infection was, at least partially, dependent on NLRP3 inflammasome activation.

In the present study, ATP was demonstrated to be beneficial for the host to fight invaded bacteria in a mouse peritonitis model. In addition, the NLRP3 inflammasome exerted an important role in the ATP-mediated facilitation of the host fight against pathogens. As a danger signal, ATP is prevalent within the body and may be quite important in initiating the immune response when the host is suffering infection. To deeply understand the detailed role and mechanism by which ATP protects the host against microbial infection, further research is required. In conclusion, we have demonstrated a protective role for ATP in fighting infection, and this role is related to NLRP3 inflammasome activation.
